# The BD FACSPresto Point of Care CD4 Test Accurately Enumerates CD4+ T Cell Counts

**DOI:** 10.1371/journal.pone.0145586

**Published:** 2015-12-31

**Authors:** Priska Bwana, Lara Vojnov, Maureen Adhiambo, Catherine Akinyi, Joy Mwende, Marta Prescott, Matilu Mwau

**Affiliations:** 1 Kenya Medical Research Institute, Nairobi, Kenya; 2 Clinton Health Access Initiative, Boston, Massachusetts, United States of America; Rush University, UNITED STATES

## Abstract

**Objective:**

Currently 50% of ART eligible patients are not yet receiving life-saving antiretroviral therapy (ART). Financial constraints do not allow most developing countries to adopt a universal test and offer ART strategy. Decentralizing CD4+ T cell testing may, therefore, provide greater access to testing, ART, and better patient management. We evaluated the technical performance of a new point-of-care CD4+ T cell technology, the BD FACSPresto, in a field methods comparison study.

**Methods:**

264 HIV-positive patients were consecutively enrolled and included in the study. The BD FACSPresto POC CD4+ T cell technology was placed in two rural health care facilities and operated by health care facility staff. We compared paired finger-prick and venous samples using the BD FACSPresto and several existing reference technologies, respectively.

**Results:**

The BD FACSPresto had a mean bias of 67.29 cells/ul and an r^2^ of 0.9203 compared to the BD FACSCalibur. At ART eligibility thresholds of 350 and 500 cells/ul, the sensitivity to define treatment eligibility were 81.5% and 77.2% and the specificities were 98.9% and 100%, respectively. Similar results were observed when the BD FACSPresto was compared to the BD FACSCount and Alere Pima. The coefficient of variation (CV) was less than 7% for both the BD FACSCalibur and BD FACSPresto. CD4+ T cell testing by nurses using the BD FACSPresto at rural health care facilities showed high technical similarity to test results generated by laboratory technicians using the BD FACSPresto in a high functioning laboratory.

**Conclusions:**

The BD FACSPresto performed favorably in the laboratory setting compared to the conventional reference standard technologies; however, the lower sensitivities indicated that up to 20% of patients tested in the field in need of treatment would be missed. The BD FACSPresto is a technology that can allow for greater decentralization and wider access to CD4+ T cell testing and ART.

## Introduction

The UNAIDS recently released a Gap Report indicating that 35 million people are infected with HIV worldwide [1]. The highest burden of HIV rests in sub-Saharan Africa with 24.7 million patients infected with HIV. Furthermore, Kenya is one of the 15 countries that account for more than 75% of the 2.1 million new HIV infections in 2013 [1]. Though significant efforts exist to end the pandemic, access to critical diagnostic testing and antiretroviral therapy (ART) is lacking. Currently, only 50% of patients eligible for life-saving ART are receiving it. Test and offer is currently not a financial or logistical feasible option in most developing countries; therefore, it will be important for programs and clinicians to ensure that the sickest patients are prioritized for receipt of ART. It is estimated that 19 million people worldwide do not currently know their HIV positive status [1]. As HIV diagnostic efforts expand so will the demand for ART. Furthermore, CD4+ T cell testing remains important for immunological and opportunistic infection management of patients with and without ART [2].

Conventional CD4+ T cell testing technologies have been the gold standard for CD4+ T cell enumeration. These technologies require constant electricity, significant infrastructure, refrigeration, and highly skilled laboratory technicians. Unfortunately, these characteristics are often very limited in most developing countries to major city centers. Significant populations of patients reside in rural areas and attend health care facilities that lack on-site CD4+ T cell testing. It will be critical to expand and decentralize CD4+ T cell testing in order to increase access to ART and ensure appropriate opportunistic infection management of patients on ART.

Point-of-care (POC) technologies are simple, easy to use technologies that can be placed near patients in health care facilities that lack the infrastructure requirements of conventional technologies. Several POC CD4+ T cell testing technologies are in the pipeline that do not require constant electricity, refrigeration, or skilled laboratory technicians [3]. The intended end-user of these technologies are lower cadres of health care facility staff using a finger-prick of blood. These technologies have the ability to revolutionize HIV diagnostic testing and patient care through bringing testing closer to the patient and allowing clinicians to make treatment decisions faster. Several studies have shown that POC CD4+ T cell testing significantly improved patient health impact through reduced test turnaround times and loss to follow-up as well as increased proportions of patients initiating ART [4,5].

The HIV program in Kenya is interested in decentralizing CD4+ T cell testing to increase patient access to this critical test for ART initiation. Previous evaluations have provided significant insight into the technical performance and operational characteristics of POC technologies to support optimal product selection in Kenya [6,7]. Prior to consideration for implementation and scale-up in Kenya, we conducted an independent technological evaluation to understand the diagnostic accuracy of the Becton Dickinson (BD) FACSPresto POC CD4 technology to several reference standards, including the BD FACSCalibur and the BD FACSCount, laboratory-based flow cytometers for CD4+ T cell enumeration.

## Materials and Methods

### Study Population

Participants were recruited between November 2014 and January 2015 at the Comprehensive Care Clinics of two health care facilities in the Busia County of Western Province, Kenya: Alupe Sub-District Hospital and Nambale Health Center. All HIV-positive patients over 18 years of age attending the selected health care facilities for treatment and care were eligible for inclusion in this study. Only patients who provided written informed consent prior to testing were enrolled in the study. This study was reviewed and approved by the Kenya Medical Research Institute Ethical Review Committee (Protocol No. 2657) and the Chesapeake Institutional Review Board (Protocol No. Pro0010512). The study was conducted in accordance with the ethical standards of the Helsinki Declaration of 1975, as revised in 2000.

### Study Design

This independent cross-sectional prospective technical methods comparison study compared the performance of the BD FACSPresto POC CD4+ T cell technology (Becton Dickinson, East Rutherford, NJ, USA) using fresh, finger-prick capillary samples collected and tested by health care facility staff with reference CD4+ T cell testing of the BD FACSCalibur, BD FACSCount (Becton Dickinson, East Rutherford, NJ, USA), and Alere Pima (Alere, Waltham, MA, USA) performed by trained laboratory technicians using matched EDTA blood. Informed consenting patients were enrolled consecutively before providing a finger-prick blood sample and a venipuncture EDTA blood sample. Qualified and trained health care facility staff, comprised of both nurses (three) and laboratory technicians (two), performed the BD FACSPresto test and drew venipuncture EDTA blood for reference laboratory CD4+ T cell testing. Demographic data and test results from each patient was collected and entered into a Microsoft Excel database. A total of 264 patients were included in the study (Table 1). One patient did not receive a BD FACSPresto result because the patient prematurely left the facility and their test resulted in an error and could not be repeated. Four patients did not receive a BD FACSCount result because of device malfunctions. Approximately 100 patient samples were randomly selected for testing on the Alere Pima in the laboratory to compare the performance of the BD FACSPresto POC CD4 technology with the currently used Alere Pima POC CD4 technology.

**Table 1:** Number of CD4+ T cell test results by technology and CD4+ T cell threshold used.

		264 total patients enrolled			
		FACSCalibur	FACSCount	FACSPresto	Pima
	Total CD4 results per technology	264	260	263	104
Number of CD4 results below or above specificied threshold	Below 100 cells/ul	20	19	15	16
	Above 100 cells/ul	244	241	248	88
	Below 350 cells/ul	82	69	68	57
	Above 350 cells/ul	182	191	195	47
	Below 500 cells/ul	146	131	112	85
	Above 500 cells/ul	118	129	151	19

Upon finger-prick using the provided lancet, blood was transferred to the BD FACSPresto cartridge by placing the finger on the open valve. The cartridge was then closed and placed in the workstation for 18 minutes of incubation at room temperature. The device display provided an interface to time the incubation period. After incubation, the cartridge sticker was removed to allow for the cell detection and cartridge inserted into the device for reading. A result print-out was produced automatically. The EDTA blood sample from each patient was delivered to the Kenya Medical Research Institute (KEMRI) laboratory for testing using the BD FACSCalibur. The laboratory operators were blinded to the BD FACSPresto results, while the health care facility staff were blinded of the reference test results. CD4+ T cell testing using both the BD FACSPresto and BD FACSCalibur were performed according to manufacturers’ instructions, by only trained staff. One hundred EDTA blood samples were used for testing the repeatability of the BD FACSPresto technology in the KEMRI laboratory using measured pipettes to apply blood to the cartridge.

For clinical management, patients were only provided with the CD4+ T cell result from the conventional reference CD4+ T cell technology. The BD FACSCalibur instrument used in this study was enrolled in and recently passed external quality assurance (EQA) schemes, including as the Western Province External Quality Assurance Scheme (WEPEQAS), CDC Inter-Laboratory EQA, and Quality Assessment and Standardization for Immunological measures relevant for HIV/AIDS programme (QASI). All laboratory technologists performing the reference CD4+ T cell technologies included in this study are trained annually in good laboratory practice, immunophenotyping for flow cytometry, and biosafety. Daily controls were run for the BD FACSCalibur and BD FACSCount laboratory-based CD4+ T cell enumeration technologies as well as for the Alere Pima and BD FACSPresto.

### Statistical Analysis Methods

The technical performance characteristics of the BD FACSPresto were analyzed using standard statistical methods for evaluating diagnostic technologies. Bland-Altman [8] and linear regression analyses were performed to determine the bias, 95% limits of agreement, and coefficient of determination (r^2^). BD FACSPresto technology repeatability was calculated on paired samples in the laboratory on the same instrument by the same technician and determined by the coefficient of variation. Finally, the sensitivity, specificity and misclassification rates of the BD FACSPresto were calculated compared with the reference CD4+ T cell technologies using the following thresholds: 100 cells/μl, used for Cryptococcal reflex testing; 350 cells/μl, the previously recommended ART initiation eligibility threshold [9]; and 500 cells/μl, the 2013 WHO recommended ART initiation eligibility threshold [10]. Clinical calculated were performed using the following standard definitions:

Sensitivity: # of patients correctly identified as below the threshold using the FACSPresto / # of patients identified as below the threshold using the reference CD4+ T cell technology x 100.
Specificity: # of patients correctly identified as above the threshold using the FACSPresto / # of patients identified as above the threshold using the reference CD4+ T cell technology x 100.

Misclassification was defined using the below equations [7]:

Upward misclassification percentage: # of patients incorrectly identified as above the threshold using the FACSPresto / # of patients identified as below the threshold using the reference CD4+ T cell technology x 100.
Downward misclassification percentage: # of patients incorrectly identified as below the threshold using the FACSPresto / # of patients identified as above the threshold using the reference CD4+ T cell technology x 100.
Positive predictive value: # of patients correctly identified as below the threshold using the FACSPresto / # of patients identified as below the threshold using the FACSPresto x 100.
Negative predictive value: # of patients correctly identified as above the threshold using the FACSPresto / # of patients identified as above the threshold using the FACSPresto x 100.

All statistical analyses were performed with GraphPad Prism™, STATA and Microsoft Excel by two independent statisticians.

## Results

A total of two hundred and sixty-four patients were enrolled in this technical accuracy evaluation. The BD FACSPresto evaluated had CE-IVD and WHO-PQ (pre-qualification) approval [11]. Approximately 75% of enrolled patients were female, while 70% of patients had a CD4+ T cell count above 350 cells/ul, as measured by the BD FACSCalibur. The mean and median ages of enrolled participants were 40 with 87% between the ages of 26 and 55 years old. Almost 70% of the BD FACSPresto tests were sampled and run by nurses. The temperature range of the health care facility where POC tests were run was between 22–32°C with a mean and median of 26°C.

Samples tested using the BD FACSCalibur had a median of 466 cells/ul (range: 5–1,776 cells/ul), compared with a median of 498 cells/ul (range: 6–1,607 cells/ul) on the BD FACSCount and a median of 563 cells/ul (range: 6–1,635 cells/ul) on the BD FACSPresto. The patient distributions by technology and CD4+ T cell threshold can be found in Table 1.

**Fig 1:**
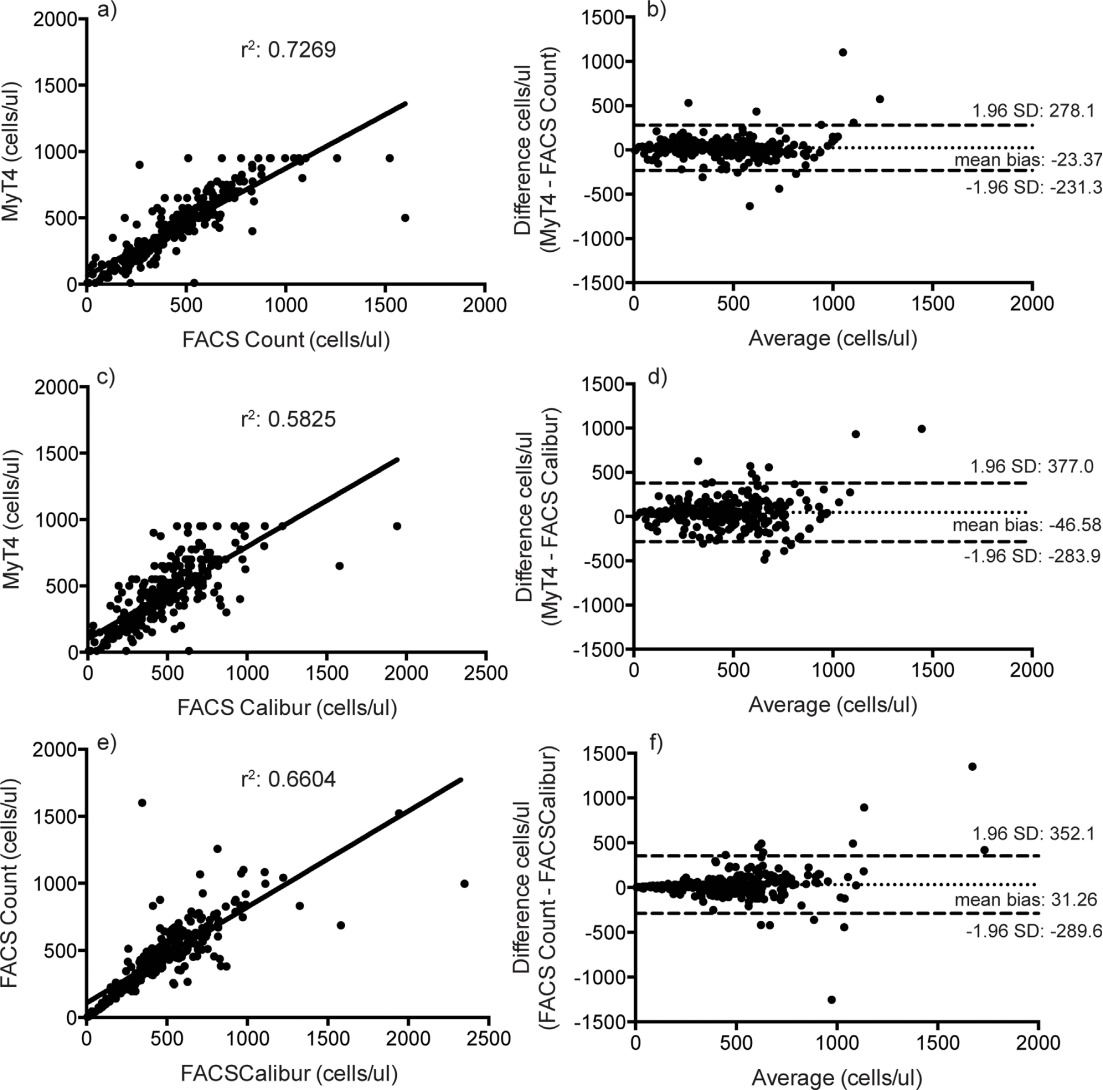
Linear regression (a, c, e) and Bland-Altman (b, d, f) analyses of absolute CD4+ T cell counts between the BD FACSPresto and BD FACSCalibur (a and b); the BD FACSPresto and BD FACSCount (c and d); and the BD FACSPresto and Alere Pima (e and f).

The BD FACSCalibur classified 69% of enrolled patients as above 350 cells/ul, while the BD FACSCount and BD FACSPresto classified 73% and 74% of enrolled patients above 350 cells/ul, respectively. The BD FACSPresto had a mean bias of 67.29, 60.41, and 48.39 and a coefficient of determination, r^2^, of 0.9203, 0.8110, and 0.8217 compared to the BD FACSCalibur, BD FACSCount, and Alere Pima, respectively (Fig 1).

Though a quantitative assay, CD4+ T cell testing in sub-Saharan Africa is often used semi-quantitatively to assess patient eligibility for reflex Cryptococcal testing (if <100 cells/ul) or ART eligibility (<350 or <500 cells/ul, depending on national guidelines). At the 100 cells/ul threshold, the sensitivity and specificity of the BD FACSPresto was 78.95% (95% confidence intervals: 54.4–98.5%) and 100.00% (98.5–100%), respectively, compared to the BD FACSCalibur (Table 2). The BD FACSPresto had a sensitivity of 81.48% (71.3–89.2%) and specificity of 98.90% (96.1–99.9%) compared to the BD FACSCalibur to correctly identify patients eligible for ART at the threshold of 350 cells/ul (Tables 2 and 3). Finally, at the 500 cells/ul ART eligibility threshold the BD FACSPresto had a sensitivity and specificity of 77.24% (69.5–83.8%) and 100.00% (96.9–100.0%), respectively (Tables 2 and 3). The sensitivity of the BD FACSPresto to correctly classify patients eligible for ART increased compared to the BD FACSCount and Alere Pima at both thresholds; however, this was unlikely to be significant given the wide confidence intervals (Table 2).

**Table 2:** Sensitivity, specificity, upward and downward misclassification rates, and positive and negative predictive values of the BD FACSPresto CD4+ T cell technology compared with the BD FACSCalibur, BD FACSCount, Alere Pima, and BD FACSPresto in the laboratory across three CD4+ T cell thresholds.

	Sensitivity (95% CI)	Specificity (95% CI)	Upward misclassification	Downward misclassification	Positive predictive value	Negative predictive value
	BD FACSCalibur					
100 cells/ul	78.9% (54.4–93.9)	100% (98.5–100)	21.1% (6.1–45.6)	0% (0–1.5)	100% (78.2–100)	98.4% (95.9–99.6)
350 cells/ul	81.5% (71.3–89.2)	98.9% (96.1–99.9)	18.5% (10.8–28.7)	1.1% (0.3–3.9)	97.1% (89.8–99.6)	92.3% (87.6–95.6)
500 cells/ul	77.2% (69.5–83.8)	100% (96.9–100)	22.8% (16.2–30.5)	0% (0–0.3)	100% (96.8–100)	78.1% (70.7–84.5)
	BD FACSCount					
100 cells/ul	72.2% (46.5–90.3)	99.6% (97.7–100)	27.8% (9.7–53.5)	0.4% (0–2.3)	92.9% (66.1–99.8)	98.0% (95.3–99.3)
350 cells/ul	83.8% (72.9–91.6)	95.3% (91.2–97.8)	16.2% (8.4–27.1)	4.7% (2.2–8.8)	86.4% (75.7–93.6)	94.3% (90.0–97.1)
500 cells/ul	80.0% (72.1–86.5)	95.3% (90.2–98.3)	20.0% (13.5–27.9)	4.7% (1.7–9.8)	94.5% (88.5–98.0)	82.6% (75.5–88.3)
	Alere Pima					
100 cells/ul	80.0% (51.9–95.7)	97.7% (92.0–99.7)	20.0% (4.3–48.1)	2.3% (0.3–8.0)	85.7% (57.2–98.2)	96.6% (90.5–99.3)
350 cells/ul	85.7% (73.8–93.6)	95.7% (85.5–99.5)	14.3% (6.4–26.2)	4.3% (0.5–14.5)	96.0% (86.3–99.5)	84.9% (72.4–93.3)
500 cells/ul	85.7% (76.4–92.4)	94.7% (74.0–99.9)	14.3% (7.6–23.6)	5.3% (0.1–26.0)	98.6% (92.6–100)	60.0% (40.6–77.3)
	BD FACSPresto in facility versus BD FACSPresto in laboratory					
100 cells/ul	54.5% (23.4–83.3)	99.2% (95.6–100)	45.5% (16.7–76.6)	0.8% (0–4.4)	85.7% (42.1–99.6)	96.1% (91.1–98.7)
350 cells/ul	88.5% (76.6–95.6)	96.4% (89.8–99.2)	11.5% (4.4–23.4)	3.6% (0.7–10.2)	93.9% (83.1–98.7)	93.0% (85.4–97.4)
500 cells/ul	89.5% (80.3–95.3)	98.3% (90.9–100)	10.5% (4.7–19.7)	1.7% (0–9.1)	98.6% (92.2–100)	87.9% (77.5–94.6)

**Table 3:** 2x2 table of the BD FACSPresto compared to the FACSCalibur in determining ART eligibility using the 350 and 500 cells/ul thresholds.

		FACSCalibur				FACSCalibur	
		<350	>350			<500	>500
FACSPresto	<350	66	2	FACSPresto	<500	112	0
	>350	15	180		>500	33	118

Patient misclassification was calculated as the proportion of patients incorrectly classified as above or below the cryptococcal testing and ART thresholds. Irrespective of the reference technology, the BD FACSPresto primarily misclassified patients as above the thresholds by up to 23%, compared with less than 6% misclassification as below these thresholds (Table 2). Compared to the BD FACSCalibur at the ART eligibility threshold of 350 cells/ul, the BD FACSPresto had a positive predictive value of 97.1% (89.8–99.6) and a negative predictive value of 92.3% (87.6–95.6). The positive and negative predictive values of the BD FACSPresto were above 80% across all thresholds for each reference technology (Table 2).

The repeatability or coefficient of variation of the BD FACSCalibur and BD FACSCount were 6.40% and 15.8%, respectively. Comparatively, the coefficient of variation of the BD FACSPresto was 6.31%.

The BD FACSPresto operated in the laboratory had a mean bias of 40.39 cells/ul and an r^2^ of 0.9153 compared to the BD FACSPresto operated in a health care clinic facility, and a mean bias of 2.704 cells/ul and an r^2^ of 0.9397 compared to the BD FACSCalibur (data not shown). At the ART eligibility thresholds of 350 and 500 cells/ul, the BD FACSPresto in the clinic facility had a sensitivity and specificity of 88% or greater compared to the BD FACSPresto in the laboratory (Table 4). Additionally, the BD FACSPresto in the laboratory had a sensitivity and specificity of 90% or greater compared to the BD FACSCalibur. The performance of the BD FACSCount and Alere Pima compared to the BD FACSCalibur was similar to previous reports [6,12,13].

**Table 4:** Sensitivity, specificity, upward and downward misclassification rates, and positive and negative predictive values of the BD FACSPresto CD4+ T cell technology used in the laboratory compared with the BD FACSCalibur across three CD4+ T cell thresholds.

	Sensitivity (95% CI)	Specificity (95% CI)	Upward misclassification	Downward misclassification	Positive predictive value	Negative predictive value
	BD FACSPresto in laboratory versus BD FACSCalibur					
100 cells/ul	63.6% (30.8–89.1)	98.4% (94.3–99.8)	36.4% (10.9–69.2)	1.6% (0.2–5.7)	77.8% (40.0–97.2)	96.8% (92.1–99.1)
350 cells/ul	92.3% (81.5–97.9)	95.2% (88.1–98.7)	7.7% (2.1–18.5)	4.8% (1.3–11.9	92.3% (81.5–97.9)	95.2% (88.1–98.7)
500 cells/ul	96.1% (88.9–99.2)	91.5% (81.3–97.2)	3.9% (0.8–11.1)	8.5% (2.8–18.7)	93.6% (85.7–97.9)	94.7% (85.4–98.9)

## Discussion

More than 10 million HIV-positive patients globally in need of life-saving ART. Further decentralization of technologies will provide greater access to CD4+ T cell testing, which remains the critical barrier to identifying patients in most need of treatment. Additionally, decentralization of CD4+ T cell testing will allow for quicker management of opportunistic infections in ART patients.

In health care facilities, the BD FACSPresto had high specificity and low downward misclassification rates meaning that the technology accurately classified almost all patients who were above the thresholds analyzed. The BD FACSPresto, however, when operated in clinic settings, had a sensitivity for diagnosing ART eligibility of between approximately 77–84% and upward misclassification rates of approximately 16–22%, depending on the reference technology used. The performance suggests that in the field the BD FACSPresto incorrectly identifies between 16–22% of sick patients as not yet sick enough to require ART. This performance would ensure that patients are not unnecessarily initiated on ART and would reduce any linked costs. However, missed patients in need of ART could result in negative patient health outcomes, morbidity, and mortality. This performance varies compared to other POC CD4 technologies that have high sensitivities to determine ART eligibility [6,7,13,14]. Given high loss-to-follow-up rates of patients in pre-ART care [4,15] and the benefits of ART treatment, it is generally more preferable to err on the side of caution and incorrectly initiate patients close to the threshold than to miss sick patients in need of ART.

Interestingly, the positive and negative predictive values of the BD FACSPresto were consistently above 85% at all thresholds analyzed compared to the reference technology tested, indicating reliable results within the population included in the study. In settings where the mean CD4+ T cell count is significantly lower than the current study population, the sensitivity and upward misclassification results should be investigated further.

When used in the laboratory, the BD FACSPresto had high sensitivity and specificity (greater than 90%) compared to the BD FACSCalibur. This improved performance over the health care facility staff could be due to less-skilled operators in the health care facilities or poor direct finger-prick blood sample application to the cartridge.

The quality of POC testing is critical to ensure accurate and reliable test results. We found that the BD FACSPresto had a test failure rate below 7% meaning that 7% of patients included in the study required a second or repeat sample and test due to either operator or device error.

The BD FACSPresto POC CD4+ T cell technology does not require constant electricity, refrigeration, or laboratory skills for operation. We found that one battery charge could last up to 6 hours, requiring overnight charging to fully recharge the battery. While this was sufficient at the health care facilities during the evaluation, this could be problematic for health care facilities lacking constant electricity overnight. Though an expiration date is provided on the test cartridge package, performing a test with an expired cartridge will not be rejected or result in an error, but provide a result. This oversight might be problematic in enabling facilities to use expired cartridges that may not provide quality test results.

This evaluation had some limitations. Interestingly, approximately 45% of patients enrolled and included in this evaluation had a CD4+ T cell count greater than 500 cells/ul. Patients were enrolled consecutively in this study indicating that the populations at these health care facilities were relatively healthy. The sample size under 100 cells/ul was unfortunately too low to draw strong conclusions on the performance for Cryptococcal reflex testing. Given the low sensitivity and high upward misclassification rates, it would be worthwhile to understand the performance of the BD FACSPresto in sicker populations of HIV-positive patients. Additionally, the study was limited in the number of patients with CD4 cell counts below 350 cells.

Given the need to expand access to CD4+ T cell testing for pre-ART patient management and replace old or broken conventional technologies, the BD FACSPresto performs well in the laboratory setting and could be considered in rational deployment of future POC CD4+ T cell technologies.

## References

[1] UNAIDS (2014) The Gap Report.

[2] Ford N, Meintjes G, Pozniak A, Bygrave H, Hill A, Peter T, et al (2015) The future role of CD4 cell count for monitoring antiretroviral therapy. The Lancet Infectious Diseases 15: 241–247. 10.1016/S1473-3099(14)70896-5 25467647

[3] UNITAID (2014) HIV/AIDS Diagnostic Technology Landscape, 4th edition.

[4] Jani IV, Sitoe NE, Alfai ER, Chongo PL, Quevedo JI, Rocha BM, et al (2011) Effect of point-of-care CD4 cell count tests on retention of patients and rates of antiretroviral therapy initiation in primary health clinics: an observational cohort study. Lancet 378: 1572–1579. 10.1016/S0140-6736(11)61052-0 21951656

[5] Wynberg E, Cooke G, Shroufi A, Reid SD, Ford N (2014) Impact of point-of-care CD4 testing on linkage to HIV care: a systematic review. J Int AIDS Soc 17: 18809 10.7448/IAS.17.1.18809 24447595 PMC3898050

[6] Mwau M, Adungo F, Kadima S, Njagi E, Kirwaye C, Abubakr N, et al (2013) Evaluation of PIMA™® Point of Care Technology for CD4 T Cell Enumeration in Kenya. PLoS ONE 8: e67612 10.1371/journal.pone.0067612 23825674 PMC3692483

[7] Mwau M, Kadima S, Mwende J, Adhiambo M, Akinyi C, Prescott M, et al (2014) Technical performance evaluation of the MyT4 point of care technology for CD4+ T cell enumeration. PLoS ONE 9: e107410 10.1371/journal.pone.0107410 25229408 PMC4167862

[8] Bland JM, Altman DG (1986) Statistical methods for assessing agreement between two methods of clinical measurement. Lancet 8: 307–310. 2868172

[9] World Health Organization (2010) Antiretroviral therapy for HIV infection in adults and adolescents. Recommendations for a public health approach: 2010 revision. 23741771

[10] World Health Organization (2013) Consolidated Guidelines on the Use of Antiretroviral Drugs for Treating and Preventing HIV Infection: Recommendations for a Public Health Approach. 24716260

[11] World Health Organization (2015) WHO list of prequalified in vitro diagnostic products. Available: http://www.who.int/diagnostics_laboratory/evaluations/PQ_list/en/

[12] Manabe Y, Wang Y, Elbireer A, Auerbach B, Castelnuovo B (2012) Evaluation of Portable Point-of-Care CD4 Counter with High Sensitivity for Detecting Patients Eligible for Antiretroviral Therapy. PLoS ONE 7: e34319 10.1371/journal.pone.0034319 22536323 PMC3334961

[13] Mtapuri-Zinyowera S, Chideme M, Mangwanya D, Mugurungi O, Gudukeya S, Hatzold K, et al (2010) Evaluation of the PIMA point-of-care CD4 analyzer in VCT clinics in Zimbabwe. J Acquir Immune Defic Syndr 55: 1–7. 10.1097/QAI.0b013e3181e93071 20622679

[14] Jani IV, Sitoe NE, Chongo PL, Alfai ER, Quevedo JI, Tobaiwa O, et al (2011) Accurate CD4 T-cell enumeration and antiretroviral drug toxicity monitoring in primary healthcare clinics using point-of-care testing. AIDS 25: 807–812. 10.1097/QAD.0b013e328344f424 21378535

[15] Fox MP, Rosen S (2015) Retention of Adult Patients on Antiretroviral Therapy in Low- and Middle-Income Countries: Systematic Review and Meta-analysis 2008–2013. J Acquir Immune Defic Syndr 69: 98–108. 10.1097/QAI.0000000000000553 25942461 PMC4422218

