# Correction: The BD FACSPresto Point of Care CD4 Test accurately enumerates CD4+ T cell counts

**DOI:** 10.1371/journal.pone.0167667

**Published:** 2016-12-09

**Authors:** Priska Bwana, Lara Vojnov, Maureen Adhiambo, Catherine Akinyi, Joy Mwende, Marta Prescott, Matilu Mwau

The incorrect image is used for Fig 1. Please see the correct Fig 1 here.

**Fig 1:**
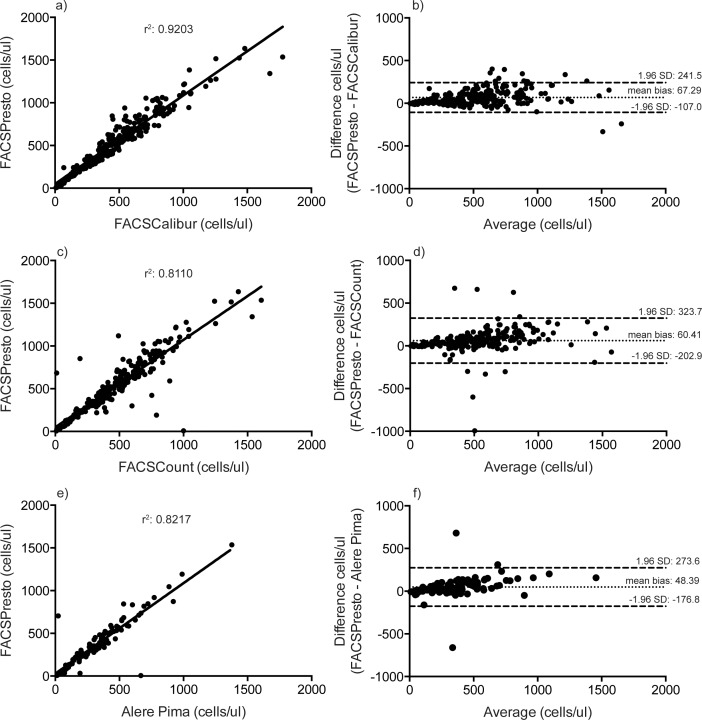
Linear regression (a, c, e) and Bland-Altman (b, d, f) analyses of absolute CD4+ T cell counts between the BD FACSPresto and BD FACSCalibur (a and b); the BD FACSPresto and BD FACSCount (c and d); and the BD FACSPresto and Alere Pima (e and f).
